# Design, Synthesis, and Cytotoxic Assessment of New Haloperidol Analogues as Potential Anticancer Compounds Targeting Sigma Receptors

**DOI:** 10.3390/molecules29112697

**Published:** 2024-06-06

**Authors:** Daniele Zampieri, Maurizio Romano, Sara Fortuna, Emanuele Amata, Maria Dichiara, Giuseppe Cosentino, Agostino Marrazzo, Maria Grazia Mamolo

**Affiliations:** 1 Department of Chemical and Pharmaceutical Sciences, University of Trieste, Via Giorgieri 1, 34127 Trieste, Italy; mamolo@units.it; 2 Department of Life Sciences, University of Trieste, Via Valerio 28, 34127 Trieste, Italy; mromano@units.it; 3 Cresset-New Cambridge House, Bassingbourn Road, Litlington, Cambridge SG8 0SS, UK; sara.fortuna@cresset-group.com; 4 Department of Drug and Health Sciences, University of Catania, Viale Doria 6, 95125 Catania, Italy; eamata@unict.it (E.A.); maria.dichiara@unict.it (M.D.); giuseppe.cosentino1996@gmail.com (G.C.); marrazzo@unict.it (A.M.)

**Keywords:** sigma 1 receptor, sigma 2 receptor, anticancer, affinity, selectivity, docking

## Abstract

Sigma receptors (SRs), including SR1 and SR2 subtypes, have attracted increasing interest in recent years due to their involvement in a wide range of activities, including the modulation of opioid analgesia, neuroprotection, and potential anticancer activity. In this context, haloperidol (HAL), a commonly used antipsychotic drug, also possesses SR activity and cytotoxic effects. Herein, we describe the identification of novel SR ligands, obtained by a chemical hybridization approach. There wereendowed with pan-affinity for both SR subtypes and evaluated their potential anticancer activity against SH-SY5Y and HUH-7 cancer cell lines. Through a chemical hybridization approach, we identified novel compounds (**4d**, **4e**, **4g**, and **4j**) with dual affinity for SR1 and SR2 receptors. These compounds were subjected to cytotoxicity testing using a resazurin assay. The results revealed potent cytotoxic effects against both cancer cell lines, with IC_50_ values comparable to HAL. Interestingly, the cytotoxic potency of the novel compounds resembled that of the SR1 antagonist HAL rather than the SR2 agonist siramesine (SRM), indicating the potential role of SR1 antagonism in their mechanism of action. The further exploration of their structure-activity relationships and their evaluation in additional cancer cell lines will elucidate their therapeutic potential and may pave the way for the development of novel anticancer agents that target SRs.

## 1. Introduction

Although sigma receptors (SRs) were initially misclassified as a class of opioid receptors, subsequent research clearly established that SRs have no homology to opioid or N-Methyl-d-Aspartate receptors (NMDARs), and they were shown to be a specific class of receptors [1]. SRs are divided into two subtypes named Sigma-1 (S1R) and Sigma-2 (S2R) [2,3,4,5]. The S1R subtype is a chaperone protein that was cloned from several tissues including tissue from humans, mice, and guinea pigs, in 1996. In 2016, A.C. Kruse et al. crystallized it, revealing a trimeric protein organization with one transmembrane domain in each protomer [6]. At the intracellular level, the S1R subtype is transported to the plasma membrane or endoplasmic reticulum, particularly within neuronal and peripheral cells. It controls the activity of some proteins (e.g., NMDARs) by regulating various ion channels through IP3-independent mechanisms [7,8].

S1Rs have significant implications for numerous functions of the central nervous system (CNS). These functions include neuroprotection, memory enhancement [9,10], the modulation of opioid pain relief [11] and involvement in drug addiction [12].

Additionally, there is evidence of increased S1R activity in certain cancers [13]. Recent findings suggest that specific S1R-targeting drugs may mitigate clinical decline in individuals who are infected with the SARS-CoV-2 virus but who are not hospitalized due to the severity of their illness [14].

In contrast, the understanding of S2R was limited until 2017, when its gene was identified as endoplasmic reticulum transmembrane protein 97 (TMEM97), also known as meningioma protein 30 (MAC-30) [15], a protein that is associated with cholesterol regulation through its interaction with the lysosomal transporter NPC1 [16,17].

The crystal structure of the S2R/TMEM97 protein was determined in 2021 [18], and several pharmacophore models have been proposed so far [19,20,21], paving the way for the discovery of new S2R ligands. S2R expression is elevated in various cancers [22,23,24,25,26,27,28], making S2R agonists promising candidates for anticancer therapy, especially in tumor types where S2R levels are high.

Given their presence in many CNS tissues, including regions implicated in movement disorders, targeting S2Rs holds promise for treating CNS conditions like neuroleptic-induced acute dystonia [29]. Furthermore, S1R antagonists and S2R agonists activators show potential in managing neuropathic pain [30,31].

Haloperidol (HAL) is a potent first-generation antipsychotic drug widely used to treat several behavioral and psychiatric conditions in adults and children. Its therapeutic effect is primarily attributed to its antagonism towards D_2_ receptors, based on the “dopamine hypothesis” [32]. The main side effects of HAL include sedation, hypotension, and extrapyramidal effects such as restlessness, tremor and stiffness. Despite its primary mode of action being dopamine antagonism, HAL also exhibits antagonist activity for NMDAR [33], as well as for S1R [34].

Furthermore, there are several publications in the literature regarding HAL derivatives with anticancer activity. In 2011, Ronsisvalle’s team studied the antitumor action of an analogue of HAL metabolite II against prostate cancer cells, demonstrating a correlation with its sigma receptor affinity [35]. In the same year, research on HAL conjugated to cationic lipid formulations was assessed, revealing its potential as a new type of anticancer therapy [36]. Later, Sozio and his group evaluated the antitumor activity of HAL metabolite II on rat C6 glioma cells and reported showing promising results [37].

Additionally, the hybridization approach has led to compounds with that have potent effects due to their dual activity involving sigma receptors [38]. Based on these observations and our ongoing efforts to discover new SR modulators, we designed and synthesized new chemical entities that were structurally related to HAL, a well-established antipsychotic drug, whose high S1R affinity (KiS1 = 2.2 nM) was also demonstrated [35]. 

**Figure 1:**
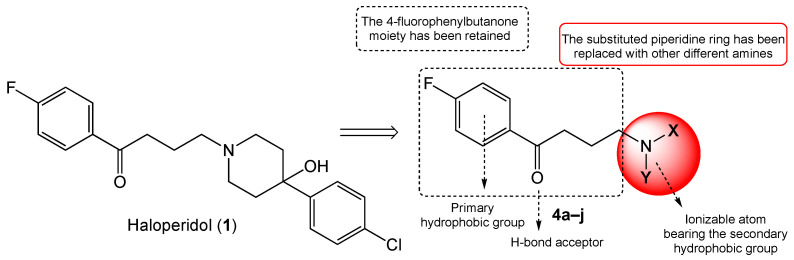
Rationale of the new HAL analogues **4a**–**j**. The 4-fluorophenylbutanone moiety has been retained to maintain the optimal distance (6–10 Å) of the primary hydrophobic group from the ionizable nitrogen atom bearing the secondary hydrophobic group, according to Glennon’s S1R pharmacophore model [39]. At the same time, it bears the carbonyl H-bond acceptor function, according to our previously published S1R pharmacophore model [40].

Specifically, we designed molecules (**4a**–**j**) (Figure 1) by retaining HAL’s original 4-fluorophenylbutan-1-one motif, which is an optimal feature in accordance with both Glennon and our S1R pharmacophore model [39,40], and by jointly replacing the 4-chlorophenyl-4-hydroxypiperidine fragment with other cyclic or linear amines found in some well-known SR ligands [41]. 

All planned compounds were synthesized, appropriately characterized and assessed to determine their affinity to both S1R and S2R through a radioligand binding assay. The best dual ligand **4g**, was computationally investigated through a molecular modeling technique which confirmed its interaction with both SR proteins. In silico ADME(T) studies of the best candidates showed good drug-likeness and a safe profile. Lastly, in vitro cytotoxicity towards two cancer cell lines was evaluated to assess the potential cytotoxicity of our new molecules.

## 2. Results and Discussion

### 2.1. Chemistry

**Scheme 1:**
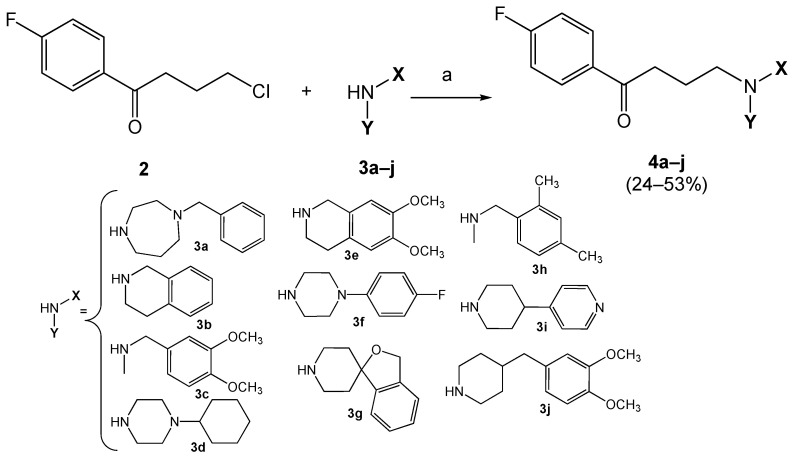
Synthesis of the new compounds **4a**–**j**. Reagent and conditions: (a) K_2_CO_3_, KI (cat), Tol, and reflux.

The hybrid compounds were synthesized following the route illustrated in Scheme 1. Compounds **4a**–**j** were obtained via N-alkylation of various commercially available amines with 4-chloro-1-(4-fluorophenyl)butan-1-one, with refluxing in toluene in basic media. All the compounds were purified via flash chromatography and properly characterized. All the spectra (Supplementary Materials) agreed with the predicted structure.

Column purification resulted in lower yields compared to the raw product, likely due to the presence of unreacted materials and unwanted products. It is worth noting that SN2 reactions can also lead to the formation of quaternary ammonium salts.

### 2.2. Biology and Computational

#### 2.2.1. SR Binding Affinities, SAR Discussion

The S1R and S2R receptor affinities of the test compounds were evaluated in competition tests by radiometric assays. The obtained affinity results for the new derivatives **4a**–**j**, in comparison with reference standards HAL (S1R antagonist), pentazocine (PTZ, S1R agonist), and *o*-ditolylguanidine (DTG, S2R agonist), are reported in Table 1.

The results of the binding affinities of the synthesized compounds revealed that derivatives **4a**, **4b**, **4i**, and **4j** showed a preference for the S1R subtype, while **4c**, **4e**, **4f**, and **4h** displayed a preference for S2R. The remaining two derivatives, **4d** and **4g**, demonstrated dual SR affinity. Among the series, the best results in terms of S1R affinity and selectivity, were achieved using 4-(3,4-dimethoxybenzyl)piperidine-based compound **4j,** showing a KiS1 of 6.1 nM and a S2/S1 ratio of 25. Regarding the best profile towards S2R, the 6,7-dimethoxy-1,2,3,4-tetrahydroisoquinoline-based compound **4e** showed the highest affinity value (KiS2 = 6.93 nM), with 241-fold higher selectivity towards the S2R than the S1R subtype. Interestingly, the simple introduction of a methylene bridge between the piperidine nucleus and the dimethoxy-substituted benzene ring in compound **4j**, compared to the tetrahydroisoquinoline moiety of derivative **4e**, shifts affinity and the selectivity from the S1R to the S2R. Another intriguing compound is derivative **4g**, which bears the 3*H*-spiro[isobenzofuran-1,4′-piperidine] fragment present in the well-known S2R agonist Siramesine (SRM). Indeed, our compound **4g** showed a slightly better profile towards both SR subtypes than SRM, with KiS1 = 6.3 nM and KiS2 = 9.2 nM, respectively (SRM, KiS1 = 10.5 nM, KiS2 = 12.6 nM)

#### 2.2.2. Cytotoxic Profiles

To gather preliminary data for later use evaluating antioxidant properties, we tested the cytotoxicity profiles of the novel compounds in human cell lines of neuroblastoma SH-SY5Y and hepatocarcinoma HUH-7, which are commonly used as neuronal models in similar studies. The results are reported in Table 2.

We selected the four best candidates from our series (compounds **4d**, **4e**, **4g**, and **4j**) to assess their cytotoxic response in the two human cancer cell lines compared to HAL, a known S1R antagonist, and SRM, a standard S2R agonist.

Cytotoxic potency was measured using a resazurin assay (see Supplementary Materials). Cells were treated with increasing concentrations of each compound for 48 hours before the cell viability was assessed (Supplementary Materials).

The cytoxicity profiles against both lines are shown in Figures S1 and S2 (Supplementary Mterials) and expressed as cytotoxic concentrations (IC_50_, Table 2).

Against the SH-SY5Y neuroblastoma cell line, the rank order of potency based on IC_50_ values was **4g** (57 μM) ≈ **4j** (58 μM) > **4e** (120 μM) >> **4d** (no measurable IC_50_). A similar pattern was observed in HUH-7 hepatocarcinoma cells, with rank a potency order of **4g** (16 μM) ≈ **4j** (37 μM) > **4e** (40 μM) >> **4d** (163 μM). These results demonstrate that the overall cytotoxicity of the compounds is comparable between the two cancer cell types.

Notably, the potency of the HAL derivatives was lower than that of the reference S2R agonist SRM (IC_50_ 5–2 μM), instead more closely resembling the S1R antagonist HAL (IC_50_ 19–41 μM). This suggests that S1R antagonism may play a prominent role in the cytotoxic mechanisms of these novel compounds.

Additionally, compounds with selectivity for S2R (**4e**) or S1R (**4j**) exhibited slightly enhanced potency against HUH-7 and SH-SY5Y cells, respectively, indicating that selectivity can tune cytotoxic effects. However, the non-selective **4g** also showed potent cytotoxicity, supporting the idea that affinity and receptor occupancy are key determinants alongside selectivity.

Overall, these results demonstrate the novel compounds have cytotoxic activity against cancer cell lines, correlating with their SR affinity and selectivity profiles.

The cytotoxicity of the HAL derivatives exhibited some distinct patterns when analyzed in the context of their SR affinity and selectivity profiles. Across both the SH-SY5Y neuroblastoma and HUH-7 hepatocarcinoma cell lines, the ranking of potency based on IC_50_ values was similar for the compounds tested. This indicates that the cytotoxic effects induced by these cancer cell types were comparable.

In fact, while the cytotoxicity data hint at some enhancement of potency by asselectivity, compound **4e** showed slightly lower IC_50_ against the HUH-7 line and **4j** did the same against SH-SY5Y cells. However, the magnitude of these differences is quite small; they are in the order of 2-fold or less between cell lines for individual compounds. Given the inherent variability in cell-based cytotoxicity assays, differences of such a small magnitude are unlikely to be biologically significant.

When compared to reference ligands, the cytotoxicity more closely resembled the S1R antagonist HAL than of the S2 agonist SRM. This suggests that S1R receptor antagonism may play a more prominent role than S2R agonism in the mechanisms of cytotoxicity for these novel compounds.

More striking is the overall comparable ranking of potency across the cell lines. The lack of major differences suggests that the cytotoxic mechanisms are not radically distinct between these cancer types. Along with the more HAL-like potency profiles, this points to S1R antagonism playing a major role across cell contexts. While tuning selectivity may fine-tune potency in a cell-dependent manner, affinity and total receptor occupancy seem to be the primary drivers of cytotoxic efficacy.

Interestingly, compound **4g**, lacking selectivity, still induced potent cytotoxicity against both cell lines, as did compounds **4e** and **4j**. This observation suggests that factors other than selectivity (including affinity and total receptor occupancy) might also influence cytotoxic potency. Therefore, we are tempted to speculate that the high dual affinity of **4g** for S1 and S2Rs may enable its cytotoxicity through extensive receptor interactions.

Further studies on an expanded panel of cancer cell lines will more fully elucidate the structure-activity relationships determining the anticancer properties of these novel haloperidol derivatives.

#### 2.2.3. Molecular Docking

**Figure 2:**
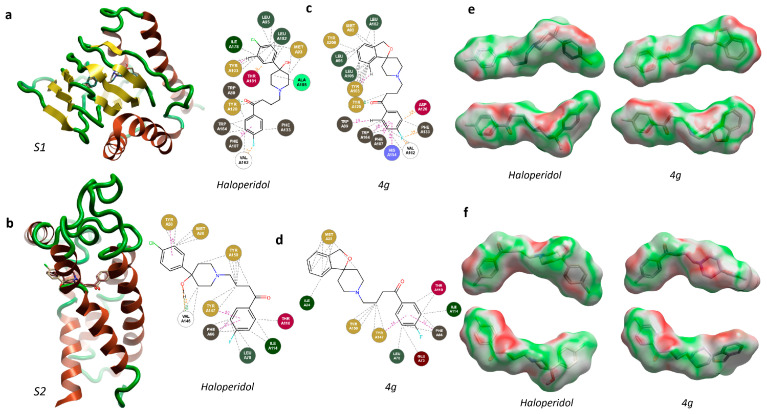
Compound **4g** and Haloperidol docking to S1R (top) and S2R (bottom): (**a**,**b**) highest-scoring three-dimensional complexes, proteins are color coded according to their structure: yellow = sheets, orange = helices, green = unstructured; **4g** and Haloperidol complexes are superposed for comparison; (**c**,**d**) comparison between **4g** (right) and Haloperidol (left) interaction maps (residues are color coded according to the RasMol ‘shapely’ color scheme, interactions are highlighted by dotted lines colour coded as follows: green = hydrogen bonds, orange = steric clashes, grey = hydrophobic contacts, purple = aromatic-aromatic); (**e**,**f**) electrostatic complementarity, with top and bottom molecular view of **4g** (right) and Haloperidol (left) in their docked poses (green = good complementarity, red = electrostatic clashes). S1R is PDB ID 5HK2 [6]; S2R was constructed by homology from PDB 7M95 [18].

Compound **4g**, which showed the best pan-affinity, and the starting HAL were docked to human models of both receptor S1R and S2R. The latter is derived by homology, as described in the methodological section. Docking lead to fairly similar docking scores. Although the observed poses differed in their geometry in the binding site (Figure 2a,b), the interactions responsible for their tight binding were of similar nature (Figure 2c,d). Both molecules were primarily bound to their target due to hydrophobic contacts with multiple residues. The fluorobenzene ring, in all cases, was kept in place by aromatic interactions (Trp89, Phe107, and Trp 164 inS1R, Phe66 and Tyr 147 in S2R). Compound **4g** also has an aromatic interaction with S1R through His 154. Overall, **4g** seems to have more contact with S1R more than it does with S2R; however, in the latter case, no steric clashes are observed while in the former instance, the large fluorine atom tends to sit too close to Phe133 and Val 162, similarly to what was originally observed for HAL, in which Val 162 clashed with the same atom in S1R. In S2R, the cyclization of the HAL-hydroxy group into a furan moiety removes the steric clash with Val 146 (Figure 2d). The electrostatic complementarity of the molecules to each receptor was also evaluated, revealing some interesting differences in this interaction pattern (Figure 2e,f). Primarily, both compounds had a good electrostatic fit with both the proteins with small unfavored spots. For S1R these were diluted on the molecule surface, with **4g** partially reducing the electrostatic clashes (Figure 2e), while for S2R, the major clash was located on the groups in proximity to the isobenzofuran oxygen but not in the oxygen itself. Moving the charges away from the oxygen while redistributing unfavorable electrostatic contributions on the adjacent rings resolves the hydroxyl-derived electrostatic clash observed in HAL.

#### 2.2.4. In Silico ADME Properties and Toxicity Prediction

We computationally predicted the drug-likeness of the same selected compounds **4d**, **4e**, **4g**, and **4j**. For this purpose, we used the SwissADME tool (www.swissadme.ch, accessed 20 November 2023, Table 3, [43]) to conduct in silico evaluation of their pharmacokinetic parameters in silico (ADME), based on an extended version of Lipinski’s rule of five (RO5) [44]. The extended version of RO5 means that orally active drugs must not violate more than one of the following requirements: MW ≤ 500, H-bond donor ≤ 5, H-bond acceptor ≤ 5, logP (related to membrane permeability) ≤ 5, logS (related to intestinal absorption) ≤ 5, and topological surface area (TPSA) ≤ 140 Å. SMILES notations were generated with Chemdraw^®^ Professional software (v. 16.0, Chembridgesoft, PerkinElmer Informatics Inc., Cambridge, MA, USA).

In contrast to HAL and SRM, which were used as reference standards, all the chosen compounds demonstrated favorable drug-like characteristics, meeting Lipinski’s rule of five with no violations. These derivatives exhibited suitable lipophilicity (cLogP around 4), facilitating their penetration of the central nervous system by efficiently crossing the blood-brain barrier (BBB). Moreover, the selected ligands displayed high gastrointestinal absorption owing to their favorable physicochemical properties, including a satisfactory solubility ranging from moderate (<4.00 mol/L) to good (>4.00 mol/L). However, they exhibited poor skin permeability (<2.5 cm/s).

Cytochrome P450 enzymes are located in the liver and intestines and are the most important enzyme species involved in drug metabolism. Drugs can inhibit or induce their action, leading to drug interactions that can cause pharmacokinetic alterations and reduce or enhance pharmacological effects. Predictions of cytochrome inhibition were calculated using a QSAR model based on an admetSAR server (http://lmmd.ecust.edu.cn/admetsar2/, accessed 24 February 2024, Table 4, [45,46]). Weconsidered six isoforms among all possible options. The results showed that all the selected compounds offer negligible inhibition, except towards CYP2C6 isoform, as is the case for reference drug HAL. Interestingly, none of the test compounds inhibit the CYP2C9 isoform, indicating a safe profile against possible bleeding complications since this isoform is involved in the metabolism of warfarin [47]. The same tool showed that common potential toxicities (carcinogenicity, Ames mutagenesis, hepatotoxicity, nephrotoxicity and more) were not detected except for acute oral toxicity and potential HERG inhibition. These were common for all the compounds used as the reference drugs (Table S1, Supplementary Materials).

## 3. Materials and Methods

### 3.1. Chemistry

#### 3.1.1. Chemical Reagents and Instruments

Unless otherwise noted, commercially available chemicals of reagent grade were used as received. TLC analyses were carried out with aluminum plates precoated with silica gel60 F254 (Merck KGaA, Darmstadt, Germany) and the visualization of spots was performed with UV light (254 nM) or iodine vapors. The silica gel (70–230 mesh, Merck KGaA, Darmstadt, Germany) was used as the stationary phase in flash column chromatography. The Stuart SMP30 (Cole-Parmer Ltd., Staffordshire, UK) apparatus was used to determine melting points (°C) and are the result were uncorrected. The FT-IR spectrophotometer Jasco 4700 (Jasco Ltd., Heckmondwike, UK) was adopted to record infrared spectra (Nujol mull). Nuclear magnetic resonance spectra were recorded using a Varian 400 MHz spectrometer (Varian Inc., Palo Alto, CA, USA), 400 MHz for ^1^H and 101 MHz for ^13^C. Chemical shifts (δ ppm) were reported, referring to CDCl_3_ (7.26 ppm for ^1^H and 77.2 ppm for ^13^C). Coupling constants (*J*) are given in Hz, with the following splitting abbreviations: s, singlet; d, doublet; dd, doublet of doublets; t, triplet; tt, triplet of triplets; tdd: triplet of doublets; q, quartet; m, multiplet; and p, pentet. Elementar Vario ELIII apparatus (Elementar UK Ltd., Stockport, UK) was employed to perform the microanalyses (C, H, N) and the results abtained agreed with the theoretical values ± 0.4%. Bruker Daltonics Esquire 4000 spectrometer (Bruker Scientific LLC, Billerica, MA, USA) was used to record the ESI-MS spectra (MeOH as solvent).

#### 3.1.2. Synthetic Procedure

##### General Synthesis of Compounds **4a**–**j**

4-(4-Benzyl-1,4-diazepan-1-yl)-1-(4-fluorophenyl)butan-1-one (**4a**)

Overall, 0.35 g of 1-benzyl-1,4-diazepane (1.0 mmol, 1 eq), 0.28 g of K_2_CO_3_ (2.0 mmol, 2 eq) and a catalytic amount of KI were added to 0.2 g of 1-(4-fluorophenyl)-butan-1-one (1.0 mmol, 1 eq) dissolved in 50 mL of toluene, and heated at reflux temperature on a magnetic stirrer-hot plate apparatus. When the reaction was completed (TLC: CH_2_Cl_2_/*n*-hexane 90:10), the mixture was washed with water (3 × 50 mL), maintaining alkalinity at pH = 9; dried over MgSO_4_; filtered; and evaporated to dryness to give a brown oil. This was then purified by flash chromatography (CH_2_Cl_2_/*n*-hexane 90:10 → CH_2_Cl_2_/EtOH 95:5) to afford pure compound **3a** as a yellow oil.

Yield: 28%. Rf: 0.3 (CH_2_Cl_2_/*n*-hexane 90:10). FT-IR (cm^−1^): 1677. ^1^H-NMR (400 MHz, CDCl_3_): δ 8.04–7.97 (m, 2H, arom.), 7.36–7.27 (m, 5H, arom.), 7.17–7.07 (m, 2H, arom.), 3.61 (s, 2H, C*H*_2_Ph), 2.96 (t, *J* = 7.1 Hz, 2H, COC*H*_2_CH_2_CH_2_N), 2.77–2.59 (m, 10H, 4×C*H*_2_ diazep. and COCH_2_CH_2_C*H*_2_N), 2.55 (t, *J* = 7.0 Hz, 2H), 1.90 (p, *J* = 7.1 Hz, 2H, COCH_2_C*H*_2_CH_2_N), 1.81–1.70 (m, 2H, NCH_2_C*H*_2_CH_2_N, diazep.). ^13^C-NMR (101 MHz, CDCl_3_): δ 198.82, 165.97 (d, C-1, *J_C_*_-*F*_ = 211.10 Hz), 139.71, 130.81 (d, C-3, C-5, *J_C_*_-*F*_ = 9.1 Hz), 128.97, 128.30, 126.96, 115.72 (d, C-2, C-6, *J_C_*_-*F*_ = 21.9 Hz, 62.90, 57.49, 55.23, 55.10, 54.54, 54.26, 36.29, 27.69, 22.54. MS-ESI: [M+H]^+^ = 355. Elemental analysis calcd (%) for C_22_H_27_FN_2_O: C 74.55, H 7.68, N 7.90; found: C 74.85, H 7.80, N 7.95.

With the same procedure, using different starting amines, compounds **4b**–**j** were obtained.

4-(3,4-Dihydroisoquinolin-2(1H)-yl)-1-(4-fluorophenyl)butan-1-one (**4b**)

Flash chromatography (CH_2_Cl_2_/*n*-hexane 90:10 → CH_2_Cl_2_/EtOH 90:10), yield: 30%. Yellow solid, Mp: 45–47 °C. Rf: 0.7 (CH_2_Cl_2_/*n*-hexane 90:10). FT-IR (cm^−1^): 1682. ^1^H-NMR (400 MHz, CDCl_3_): δ 8.04–7.94 (m, 2H, arom.), 7.22–7.90 (m, 6H, arom.), 7.08–7.00 (m, 1H, arom.), 3.79 (s, 2H, C*H*_2_ (H_1,1′_) isoquin.), 3.10 (m, 2H, COC*H*_2_CH_2_CH_2_N), 2.93 (dd, *J* = 14.9, 5.2 Hz, 4H, H_3,3′_–H_4,4′_ isoquin.), 2.81–2.70 (t, *J* = 7.1 Hz, 2H, COCH_2_CH_2_C*H*_2_N), 2.01 (tt, *J* = 6.9, 6.1 Hz, 2H, COCH_2_C*H*_2_CH_2_N). ^13^C-NMR (101 MHz, CDCl_3_): 13C NMR (101 MHz, cdcl3) δ 198.24, 165.70 (d, C-1, *J_C_*_-*F*_ = 254.4 Hz), 133.49, 130.67 (d, C-3, C-5, *J_C_*_-*F*_ = 9.4 Hz), 130.67, 130.62, 128.69, 126.64, 126.01, 115.62 (d, C-2, C-6, *J_C_*_-*F*_ = 22.0 Hz), 115.57, 115.51, 56.69, 55.31, 50.43, 35.94, 26.85, 21.01. MS-ESI: [M+H]^+^ = 298. Elemental analysis calcd (%) for C_19_H_20_FNO: C 76.74, H 6.78, N 4.71; found: C 76.85, H 6.50, N 4.55.

4-((3,4-Dimethoxybenzyl)(methyl)-amino)-1-(4-fluorophenyl)butan-1-one (**4c**)

Flash chromatography (CH_2_Cl_2_/*n*-hexane 80:20 → CH_2_Cl_2_/EtOH 95:5), yield: 35%. Yellow oil. Rf: 0.7 (CH_2_Cl_2_/EtOH 90:10). FT-IR (cm^−1^): 1687. ^1^H-NMR (400 MHz, CDCl_3_): δ 8.01–7.91 (m, 2H, arom.), 7.16–7.06 (m, 2H, arom.), 6.99 (d, *J* = 1.9 Hz, 1H, arom.), 6.87–6.74 (m, 2H, arom.), 3.86 (s, 3H, OCH_3_), 3.85 (s, 3H, OC*H*_3_), 3.58 (d, *J* = 11.7 Hz, C*H*_2_Ph), 3.02 (t, *J* = 7.0 Hz, 2H, COC*H*_2_CH_2_CH_2_N), 2.59 (t, *J* = 7.2 Hz, 2H, COCH_2_CH_2_C*H*_2_N), 2.33 (s, 3H, NC*H*_3_), 2.02 (p, *J* = 7.0 Hz, 2H, COCH_2_C*H*_2_CH_2_N). ^13^C-NMR (101 MHz, CDCl_3_): δ 198.09, 165.83 (d, C-1, *J_C_*_-*F*_ = 254.8 Hz), 149.05, 148.57, 133.31, 130.74 (d, C-3, C-5, *J_C_*_-*F*_ = 9.3 Hz), 121.69, 115.78 (d, C-2, C-6, *J_C_*_-*F*_ = 21.9 Hz), 112.47, 110.77, 61.58, 55.96, 55.86, 55.82, 41.46, 35.92, 20.91. MS-ESI: [M+H]^+^ = 346. Elemental analysis calcd (%) for C_20_H_24_FNO_3_: C 69.55, H 7.00, N 4.06; found: C 69.83, H 7.06, N 4.25.

4-(4-Cyclohexylpiperazin-1-yl)-1-(4-fluorophenyl)butan-1-one (**4d**)

Flash chromatography (CH_2_Cl_2_/*n*-hexane 80:20 → CH_2_Cl_2_/EtOH 90:10), yield: 32%. Yellow solid, Mp: 198–200 °C. Rf: 0.2 (CH_2_Cl_2_/EtOH 90:10). FT-IR (cm^−1^): 1683. ^1^H-NMR (400 MHz, CDCl_3_): δ 8.02–7.94 (m, 2H, arom.), 7.13 (t, *J* = 8.6 Hz, 2H, arom.), 2.96 (t, *J* = 6.8 Hz, 2H, COC*H*_2_CH_2_CH_2_N), 2.86 (m, 6H, 3×C*H*_2_ pip.), 2.53 (t, *J* = 7.3 Hz, 2H, COCH_2_CH_2_C*H*_2_N), 2.08 (s, 1H, NC*H* cyclohex.), 1.98 (t, *J* = 7.0 Hz, 2H COCH_2_C*H*_2_CH_2_N), 1.87 (d, *J* = 12.5 Hz, 2H, C*H*_2_ cyclohex.), 1.67 (d, *J* = 13.2 Hz, 2H, C*H*_2_, cyclohex.), 1.44–1.05 (m, 8H, C*H*_2_ cyclohex.). ^13^C-NMR (101 MHz, CDCl_3_): δ 197.85, 165.64 (d, C-1, *J_C_*_-*F*_ = 254.8 Hz), 133.51, 130.58 (d, C-3, C-5, *J_C_*_-*F*_ = 9.3 Hz), 115.68 (d, C-2, C-6, *J_C_*_-*F*_ = 21.8 Hz), 64.92, 56.90, 50.34, 48.01, 35.83, 27.23, 25.30, 25.21, 21.28. MS-ESI: [M+H]^+^ = 333. Elemental analysis calcd (%) for C_20_H_29_FN_2_O: C 72.25, H 8.79, N 8.43; found: C 72.05, H 8.60, N 8.54.

4-(6,7-Dimetoxy-3,4-dihydroisoquinolin-2(1H)-yl)-1-(4-fluorophenyl)butan-1-one (**4e**)

Flash chromatography (CH_2_Cl_2_/*n*-hexane 90:10 → CH_2_Cl_2_/EtOH 95:5), yield: 53%. Dark yellow oil, Rf: 0.4 (CH_2_Cl_2_/EtOH 90:10). FT-IR (cm^−1^): 1689. ^1^H-NMR (400 MHz, CDCl_3_): δ 8.04–7.93 (m, 2H, arom.), 7.16–7.04 (m, 2H, arom.), 6.58 (s, 1H, arom.), 6.51 (s, 1H, arom.), 3.82 (s, 3H, OC*H*_3_), 3.83 (s, 3H, OCH_3_), 3.58 (s, 2H, C*H*_2_ (H_1,1′_) isoquin.), 3.05 (t, *J* = 7.1 Hz, 2H, COC*H*_2_CH_2_CH_2_N), 2.84–2.70 (m, 4H, 2×C*H*_2_ (H_3,3′–4,4′_) isoquin.), 2.61 (t, *J* = 7.0 Hz, 2H, COCH_2_CH_2_C*H*_2_N), 2.05 (p, *J* = 7.0 Hz, 2H, COCH_2_C*H*_2_CH_2_N). ^13^C-NMR (101 MHz, CDCl_3_): δ 198.54, 165.62 (d, C-1, *J_C_*_-*F*_ = 254.3 Hz), 147.59, 147.26, 133.52, 130.65 (d, C-3, C-5, *J_C_*_-*F*_ = 9.2 Hz), 126.12, 125.99, 115.55 (d, C-2, C-6, *J_C_*_-*F*_ = 21.8 Hz), 111.33, 109.44, 57.06, 55.92, 55.90, 55.41, 50.80, 36.10, 28.35, 21.56. MS-ESI: [M+H]^+^ = 358. Elemental analysis calcd (%) for C_21_H_24_FNO_3_: C 70.57, H 6.77, N 3.92; found: C 70.35, H 6.60, N 3.90.

1-(4-Fluorophenyl)-4-(4-(4-fluorophenyl)piperazin-1-yl)butan-1-one (**4f**)

Flash chromatography (CH_2_Cl_2_ 100 → CH_2_Cl_2_/EtOH 95:5), yield: 45%. Yellow solid, Mp: 94–95 °C. Rf: 0.5 (CH_2_Cl_2_/EtOH 90:10). FT-IR (cm^−1^): 1681. ^1^H-NMR (400 MHz, CDCl_3_): δ 8.05–7.95 (m, 2H, arom.), 7.18–7.07 (m, 2H, arom.), 7.00–6.90 (m, 2H, arom.), 6.90–6.80 (m, 2H, arom.), 3.14–3.04 (m, 4H, 2×C*H*_2_ piperaz.), 3.00 (t, *J* = 7.1 Hz, 2H, COC*H*_2_CH_2_CH_2_N), 2.64–2.57 (m, 4H, 2×C*H*_2_ piperaz.), 2.48 (t, *J* = 7.1 Hz, 2H, COCH_2_CH_2_C*H*_2_N), 1.99 (p, *J* = 7.1 Hz, 2H, COCH_2_C*H*_2_CH_2_N). ^13^C-NMR (101 MHz, CDCl_3_): δ 198.37, 165.64 (d, C-1, *J_C_*_-*F*_ = 254.4 Hz), 157.13 (d, C-1′, *J_C_*_-*F*_ = 238.6 Hz), 147.93 (C-4′, d, *J_C_*_-*F*_ = 2.2 Hz), 133.59, 133.56, 130.65 (d, C-3, C-5, *J_C_*_-*F*_ = 9.3 Hz), 117.74 (d, C-2′, C-6′, *J_C_*_-*F*_ = 7.6 Hz), 115.61 (d, C-3′, C-5′, *J_C_*_-*F*_ = 21.8 Hz), 115.47 (d, C-2, C-6, *J_C_*_-*F*_ = 22.1 Hz), 57.59, 53.08, 50.01, 36.11, 21.46. MS-ESI: [M+H]^+^ = 345. Elemental analysis calcd (%) for C_20_H_22_F_2_N_2_O: C 69.75, H 6.44, N 8.13; found: C 69.85, H 6.40, N 8.24.

1-(4-Fluorophenyl)-4-(3H-spiro[isobenzofuran-1,4′-piperidin]-1′-yl)butan-1-one (**4g**)

Flash chromatography (CH_2_Cl_2_ 100 → CH_2_Cl_2_/EtOH 95:5), yield: 25%. Dark yellow solid Mp: 88–90 °C; Rf: 0.45 (CH_2_Cl_2_/EtOH 90:10). FT-IR (cm^−1^): 1684. ^1^H-NMR (400 MHz, CDCl_3_): δ 8.03–7.93 (m, 2H, arom.), 7.29–7.21 (m, 2H, arom.), 7.24–7.13 (m, 2H, arom.), 7.17–7.04 (m, 2H, arom.), 5.03 (s, 2H, C*H*_2_ fur.), 3.27–3.18 (m, 2H, C*H*_2_, pip.), 3.11 (t, *J* = 6.8 Hz, 2H, COC*H*_2_CH_2_CH_2_N), 2.90–2.79 (m, 4H, COCH_2_CH_2_C*H*_2_N and CH_2_ pip.), 2.35 (td, *J* = 13.5, 4.3 Hz, 2H, COCH_2_C*H*_2_CH_2_N), 2.22–2.10 (m, 2H, C*H*_2_ pip.), 1.83–1.74 (m, 2H, C*H*_2_ pip.). ^13^C-NMR (101 MHz, CDCl_3_): δ 197.41, 165.76 (d, C-1, *J_C_*_-*F*_ = 254.9 Hz), 143.94, 138.45, 133.12, 133.09, 130.71 (d, C-3, C-5, *J_C_*_-*F*_ = 9.3 Hz), 128.04, 127.64, 121.09, 120.99, 115.72 (d, C-2, C-6, *J_C_*_-*F*_ = 21.9 Hz), 83.20, 71.06, 57.25, 49.79, 35.84, 34.76, 19.74. MS-ESI: [M+H]^+^ = 354 *m*/*z*. Elemental analysis calcd (%) for C_22_H_24_FNO_2_: C 74.76, H 6.84, N 3.96; found: C 75.07, H 6.92, N 3.84.

4-((2,4-Dimethylbenzyl)(methyl)amino)-1-(4-fluorophenyl)butan-1-one (**4h**)

Flash chromatography (CH_2_Cl_2_ 100 → CH_2_Cl_2_/EtOH 98:2), yield: 38%. Yellow oil. Rf: 0.4 (CH_2_Cl_2_/EtOH 90:10). FT-IR (cm^−1^): 1686. ^1^H-NMR (400 MHz, CDCl_3_): δ 8.00–7.90 (m, 2H, arom.), 7.16–7.07 (m, 3H, arom.), 6.93 (s, 2H, arom.), 3.42 (s, 2H, C*H*_2_Ph), 2.96 (t, *J* = 7.2 Hz, 2H, COC*H*_2_CH_2_CH_2_N), 2.46 (t, *J* = 6.8 Hz, 2H, COCH_2_CH_2_C*H*_2_N), 2.29 (ds, 6H, 2×CH_3_ arom.), 2.19 (s, 3H, NC*H*_3_), 1.92 (p, *J* = 7.0 Hz, 2H, COCH_2_C*H*_2_CH_2_N). ^13^C-NMR (101 MHz, CDCl_3_): δ 198.66, 165.61 (d, C-1, *J_C_*_-*F*_ = 254.1 Hz), 137.10, 136.50, 134.04, 133.56, 133.53, 131.07, 130.59 (d, C-3, C-5, *J_C_*_-*F*_ = 9.3 Hz), 129.93, 126.10, 115.53 (d, C-2, C-6, *J_C_*_-*F*_ = 21.8 Hz), 60.23, 56.50, 41.88, 36.02, 21.78, 20.97, 19.12. MS-ESI: [M+H]^+^ = 314 *m*/*z*. Elemental analysis calcd (%) for C_20_H_24_FNO: C 76.65, H 7.72, N 4.47; found: C 76.77, H 7.90, N 4.34.

1-(4-Fluorophenyl)-4-(4-(pyridin-4-yl)piperidin-1-yl)butan-1-one (**4i**)

Flash chromatography (CH_2_Cl_2_ 100 → CH_2_Cl_2_/EtOH 90:10), yield: 24%. Dark yellow solid, Mp: 102–104. Rf: 0.2 (CH_2_Cl_2_/EtOH 90:10). FT-IR (cm^−1^): 1686. ^1^H-NMR (400 MHz, CDCl_3_): δ 8.52–8.46 (m, 4H, arom.), 8.06–7.96 (m, 2H, arom.), 7.18–7.08 (m, 4H, arom.), 3.11 (d, *J* = 11.3 Hz, 2H, C*H*_2_, pip.), 3.01 (t, *J* = 7.0 Hz, 2H, COC*H*_2_CH_2_CH_2_N), 2.70 (s, 1H), 2.57–2.47 (m, 3H, COCH_2_CH_2_C*H*_2_N e CH pip.), 2.15 (td, *J* = 11.6, 2.9 Hz, 2H, CH_2_, pip.), 2.02 (p, *J* = 7.1 Hz, 2H, COCH_2_C*H*_2_CH_2_N), 1.80 (tdd, *J* = 16.1, 11.1, 3.3 Hz, 4H, 2×C*H*_2_, pip.). ^13^C-NMR (101 MHz, CDCl_3_): δ 198.18, 165.67 (d, C-1, *J_C_*_-*F*_ = 254.5 Hz), 154.56, 149.87, 133.48, 130.67 (d, C-3, C-5, *J_C_*_-*F*_ = 9.1 Hz), 122.27, 115.64 (d, C-2, C-6, *J_C_*_-*F*_ = 21.7 Hz), 57.81, 53.71, 41.65, 36.11, 32.04, 21.35. MS-ESI: [M+H]^+^ = 327 *m*/*z*. Elemental analysis calcd (%) for C_20_H_23_FN_2_O: C 73.59, H 7.10, N 8.58; found: C 73.70, H 6,95, N 8.50.

4-(4-(3,4-Dimetoxybenzyl)piperidin-1-yl)-1-(4-fluorophenyl)butan-1-one (**4j**)

Flash chromatography (CH_2_Cl_2_ 100 → CH_2_Cl_2_/EtOH 90:10), yield: 34%. Yellow oil. Rf: 0.2 (CH_2_Cl_2_/EtOH 90:10). FT-IR (cm^−1^): 1679. ^1^H-NMR (400 MHz, CDCl_3_): δ 7.99–7.91 (m, 2H, arom.), 7.09 (t, *J* = 8.6 Hz, “H, arom.), 6.75 (d, *J* = 8.0 Hz, 1H, arom.), 6.67–6.59 (m, 2H, arom.), 3.82 (ds, 6H, 2×OC*H*_3_), 3.23 (d, *J* = 11.2 Hz, 2H, COC*H*_2_CH_2_CH_2_N), 3.07 (t, *J* = 6.7 Hz, 2H, CH_2_ pip.), 2.73 (t, *J* = 7.6 Hz, 2H, CH_2_ pip.), 2.48 (d, *J* = 5.8 Hz, 2H, C*H*_2_Ph), 2.30 (t, *J* = 10.7 Hz, 2H, COCH_2_CH_2_C*H*_2_N), 2.10 (m, *J* = 6.9 Hz, 2H, COCH_2_C*H*_2_CH_2_N), 1.81–1.49 (m, 5H, 2×C*H*_2_ and C*H* pip.). ^13^C-NMR (101 MHz, CDCl_3_): δ 197.60, 165.77 (d, C-1, *J_C_*_-*F*_ = 255.0 Hz), 148.74, 147.34, 133.07, 133.04, 132.37, 130.69 (d, C-3, C-5, *J_C_*_-*F*_ = 9.3 Hz), 120.90, 115.71 (d, C-2, C-6, *J_C_*_-*F*_ = 21.8 Hz), 112.26, 111.14, 105.98, 57.10, 55.88, 55.83, 53.44, 53.19, 42.02, 37.16, 35.85, 30.09, 19.72. MS-ESI: [M+H]^+^ = 400 *m*/*z*. Elemental analysis calcd (%) for C_24_H_30_FNO_3_: C 72.16, H 7.57, N 3.51; found: C 72.17, H 7.52, N 3.14.

### 3.2. Computational

#### Docking

S1R was modelled by homology by using chain A from PDB 5HK2 as a template [6]. Further details can be found in Ref. [41]. The human S2R was constructed based on homology by employing the corresponding bovine receptor as a structural template from PDB 7M95 [18], as performed in Ref. [48]. Ligands were minimized with OpenMM and the AM1-BCC method. Docking was as implemented in Flare Essentials V8 by using the “Very Accurate but Slow” method and by centering a 6.0Å docking grid on the crystallographic ligands. In both cases, the optimum pose was selected for comparison. Contact maps and electrostatic complementarities were calculated with Flare™ default parameters. Calculations were run and images generated with Flare™, version 8.0, Cresset^®^, Litlington, Cambridgeshire, UK; http://www.cresset-group.com/flare/ [49,50,51].

### 3.3. Biology

#### 3.3.1. S1R and S2R Binding Assays

Liver homogenates were prepared from Sprague Dawley rats (ENVIGO RMS S.R.L., Udine, Italy) for both S1R and S2R receptor binding assays. We purchased [^3^H]-Pentazocine (26.9 Ci/mmol) and [^3^H]1,3-di-*o*-tolylguanidine ([^3^H]DTG, 35.5 Ci/mmol) from PerkinElmer (Zaventem, Belgium). An Ultima Gold MV Scintillation cocktail was sourced from PerkinElmer (Milan, Italy). Other materials were obtained from Merck Life Science S.r.l. (Milan, Italy). For each compound, a10 mM DMSO stock solution was obtained. From these, diluted test concentrations ranging from 10^−5^ M to 10^−11^ M wereprepared using the respective assay buffer [52]. Both assays involve the use of a tris buffer (50 mM, pH 8) and liver homogenates (250 μg/sample) from male Sprague Dawley rats [53]. During in vitro S1R ligand binding assays, [^3^H]-pentazocine (2 nM; K_d_ = 2.9 nM) was employed as a radioligand and non-specific binding was measured using unlabeled (+)-pentazocine (10 μM). In vitro S2R ligand binding assays were performed, using [^3^H]-DTG (2 nM; K_d_ = 17.9 nM) as a radioligand, (+)-pentazocine (5 μM) as S1R masking agent, and DTG (10 μM) for the measurement of non-specific binding. The final volume was 0.5 mL [54]. A millipore filter apparatus was employed to separate bound and free radioligands via filtration under conditions of reduced pressure through Whatman GF 6 glass fiber filters. Each filter paper was washed three times with 2 mL of ice-cold tris buffer (50 mM for S1R, 10 mM for S2R; pH 8.0), dried at room temperature, and incubated overnight with 2 mL of Ultima Gold MV Scintillation cocktail in pony vials. Bound radioactivity was determined using a liquid scintillation counter Beckman LS 6500 (Beckman Coulter Diagnostic, Brea, CA, USA. GraphPad Prism^®^ 7.0 (GraphPad Software, San Diego, CA, USA) was employed to calculate the K_i_-values that were presented as mean values ± SD from at least two independent experiments conducted in duplicate.

#### 3.3.2. Viability Assays

Dulbecco’s modified Eagle medium (DMEM; Gibco, Life Technologies, Grand Island, NY, USA), supplemented with 10% (*v*/*v*) of fetal bovine serum (FBS) and Antibiotic Antimycotic Solution (100 U penicillin, 100 μg/mL streptomycin and 0.25 μg/mL amphotericin B; Sigma-Aldrich, St. Louis, MO, USA), was used to culture the human cell lines SH-SY5Y [55] (neuroblastoma, ATCC, CRL-2266) and HUH-7 [56] (hepatocarcinoma, Japanese Cancer Research Resources Bank, National Institutes of Biomedical Innovation, Health and Nutrition, NIBIOHN, JCRB0403).

Cell viability was evaluated using resazurin (SERVA Electrophoresis GmbH, Heidelberg, Germany) as previously described [57].

Briefly, a resazurin stock solution (440 μM, 10× in phosphate-buffered saline) was diluted in a culture medium to prepare a 44 μM working solution. Cells (5 × 10^3^) were seeded in 96-well plates, incubated with 100µl of resazurin working solution, and fluorescence was measured after 1, 2, and 3 hrs. The background fluorescence of killed cells was subtracted, and viability was calculated relative to control cells treated with DMSO solvent (1%). The fluorescence was measured using an Envision 2104 Multi-label Microplate Reader (Perkin Elmer, Boston, MA, USA) and by setting the excitation wavelength at λ = 530 nm and emission wavelength at λ = 590 nm. Measurements were performed at a time of 0 and after 1 hour of incubation. Cytotoxicity was calculated as 100% minus the percentage of viability.

Statistical analysis was carried out with GraphPad Prism^®^ 6.0 software. An unpaired *t*-test was used to compare treated and control cells, with *p* < 0.05 considered statistically significant. The half-maximal inhibitory concentration (IC_50_) values for cytotoxicity were determined by analyzing dose-response curves using GraphPad Prism software.

## 4. Conclusions

To find novel SR modulators with potential anticancer activity, we synthesized a series of novel haloperidol analogues by merging hybridization approaches. Indeed, we kept the original 4-fluorophenylbutan-1-one motif of HAL, and jointly replaced the 4-chlorophenyl-4-hydroxypiperidine fragment with other cyclic or linear amines present in some well-known SR ligands.

Notably, compound **4g**, which displayed the strongest pan-affinity for both the S1R and S2R subtypes, was particularly effective at inducing cytotoxicity in these cancer cell lines.

The high dual affinity of compound **4g** for both S1R and S2R may enable extensive receptor interactions, potentially contributing to its observed cytotoxic potency. This finding suggests that factors beyond just receptor selectivity, such as overall receptor binding affinity and occupancy, can influence the anticancer activity of these compounds.

Interestingly, the cytotoxic potency of the novel compounds more closely resembled that of the S1R antagonist HAL, rather than the S2R agonist SRM, hinting at a potential role of S1R antagonism in their mechanism of action. This insight highlights the therapeutic potential of targeting S1R in the development of new anticancer agents.

Additionally, the favorable ADME(T) properties predicted for these novel haloperidol analogues, including drug-likeness, solubility, permeability, and low toxicity profiles, further support their potential for continued preclinical and clinical investigation as promising anticancer drug candidates.

Overall, our study highlights the successful design and synthesis of novel HAL analogues with dual SR1 and SR2 affinity with potent cytotoxic effects against cancer cell lines. The integration of SR binding affinities, SAR analysis, molecular docking, cytotoxic profiles, and in silico ADME and toxicity prediction provides a comprehensive understanding of the potential therapeutic applications of these compounds and paves the way for their further development as novel anticancer agents targeting SRs.

## Figures and Tables

**Table 1:** Binding affinities towards S1R and S2R, and selectivity ratios for compounds **4a**–**j**.

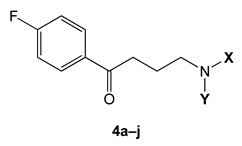					
Cmpd	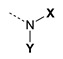	Ki S1R (nM) ^a^	Ki S2R (nM) ^a^	S2R/S1R	S1R/S2R
**4a**	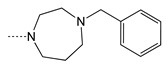	21.58 ± 3.44	210.38 ± 21.37	10	0.5
**4b**	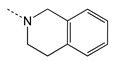	44.46 ± 7.39	124.17 ± 16.27	2.8	0.35
**4c**	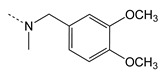	301.30 ± 74.73	148.25 ± 10.34	0.5	2.0
**4d**	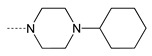	18.79 ± 2.91	13.15 ± 2.78	0.7	1.4
**4e**	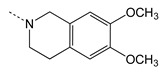	1663.41 ± 118.09	6.93 ± 0.76	0.004	241
**4f**	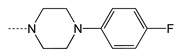	70.15 ± 23.19	16.37 ± 3.17	0.23	4.3
**4g**	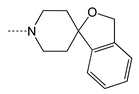	6.30 ± 1.40	9.23 ± 0.95	1.46	0.7
**4h**	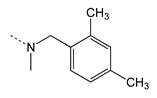	59.70 ± 7.60	27.73 ± 3.03	0.46	2.1
**4i**	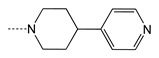	13.90 ± 3.11	28.31 ± 3.33	2.03	0.5
**4j**	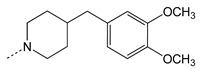	6.10 ± 0.58	152.76 ± 30.31	25	0.04
HAL ^b^	-	2.2 ± 0.5	16 ± 1.7	30	0.03
(+)-PTZ ^c^	-	4.3 ± 0.5	1465 ± 224	312	0.003
DTG ^d^	-	124 ± 19	18 ± 1	0.14	6.9
SRM ^e^	-	10.5 ± 2.6	12.6 ± 0.1	1.2	0.8

^a^ Each value is the mean ± SEM of at least two experiments performed in triplicate (SRs); ^b^ haloperidol (data from [35]); ^c^ pentazocine; ^d^ *o*-ditolylguanidine; ^e^ siramesine (data from [42]).

**Table 2:** IC_50_ values (µM) of the novel SR ligands **4d**, **4e**, **4g**, and **4j** compared to HAL and SRM controls in SH-SY5Y and HUH7 cell lines. Cells were treated for 48 hrs with various concentrations of the indicated compounds before evaluating cell viability through a resazurin assay. Data are expressed as mean ± SEM of three independent experiments performed in triplicate. Lower IC_50_ values indicate higher cytotoxicity.

Cmpd	IC_50_ (μM)	
	SH-SY5Y	HUH-7
**4d**	0	163 ± 18
**4e**	120 ± 13	40 ± 5
**4g**	57 ± 6	16 ± 2
**4j**	58 ± 7	37 ± 6
HAL	41 ± 6	19 ± 2
SRM	5.0 ± 1	2.0 ± 0.25

**Table 3:** In silico prediction of the main pharmacokinetic parameters of the test compounds **4d**, **4e**, **4g** and **4j**. Data are reported for reference compounds HAL (S1R antagonist), and SRM (S2R agonist) for comparison.

Cmpd	clogP	clogS	Skin Permeant	RO5 Violation	BBB Permeant	GI Abs.
	[C*_octanol_*]/[C*_water_*]	(mol/L)*_water_*	cm/s	≤1	-	-
**4d**	3.64	−4.00	−5.73	0	Yes	High
**4e**	3.72	−4.15	−5.99	0	Yes	High
**4g**	3.91	−4.12	−6.11	0	Yes	High
**4j**	4.54	−4.98	−5.41	0	Yes	High
HAL	4.22	−4.82	−5.54	0	Yes	High
SRM	5.85	−6.52	−4.78	1	No	Low

**Table 4:** In silico prediction of potential cytochrome inhibition of the test compounds **4d**, **4e**, **4g** and **4j**. Data are reported for reference compounds HAL (S1R antagonist), and SRM (S2R agonist) for comparison.

Cmpd	CYP2C8	CYP1A2	CYP2C19	CYP2C9	CYP2D6	CYP3A4
	Inhibition					
**4d**	−0.8113	+0.6893	+0.5571	−0.7488	+0.8636	−0.8616
**4e**	−0.5596	−0.5000	−0.6131	−0.7220	+0.6847	−0.8254
**4g**	−0.7173	−0.6239	−0.7124	−1.0000	+0.5507	−0.6430
**4j**	+0.7192	−0.7398	−0.6508	−0.6607	+0.6378	−0.8955
HAL	−0.6600	−0.9045	−0.9248	−1.0000	+0.5101	+0.6899
SRM	−0.7403	+0.7752	+0.5786	−0.7062	−0.5673	+0.5827

## Data Availability

The data presented in this study are available on request from the corresponding author.

## Supplementary Materials

The following supporting information can be downloaded at: https://www.mdpi.com/article/10.3390/molecules29112697/s1, Figure S1: Competition curves for compounds **4d**, **4e**, **4g**, and **4j** in comparison with HAL and SRM in SH-SY5Y cells. Figure S2: Competition curves for compounds **4d**, **4e**, **4g**, and **4j** in comparison with HAL and SRM in HUH-7 cells. Table S1: main predicted toxicity for compounds **4d**, **4e**, **4g**, and **4j** in comparison with HAL and SRM.
