# Supplementary material for: Design, Synthesis, and Cytotoxic Assessment of New Haloperidol Analogues as Potential Anticancer Compounds Targeting Sigma Receptors
Source: Molecules. 2024 Jun 6;29(11):2697. doi: 10.3390/molecules29112697 (PMC11173765; doi:10.3390/molecules29112697)
Supplement: Supplementary file 1 [file molecules-29-02697-s001.zip › molecules-3011438-supplementary.pdf]

Supplementary Material

Design, Synthesis, and Cytotoxic Assessment of Hew Haloperidol Analogues as Potential Anticancer Compounds Targeting Sigma Receptors

D. Zampieri[1*], M. Romano[2], S. Fortuna[3], E. Amata[4], M. Dichiara[4], A. Marrazzo[4] and M.G. Mamolo[1]

[1] *Department of Chemical and Pharmaceutical Sciences, University of Trieste, Via Giorgieri 1, 34127 Trieste, Italy*

[2] *Department of Life Sciences, University of Trieste, Via Valerio 28/1, 34127 Trieste, Italy*

[3] *Italian Institute of Technology (IIT), Via E. Melen 83, 16152 Genova, Italy*

[4] *Department of Drug and Health Sciences, University of Catania, Viale Doria 6, 95125 Catania, Italy*

* Corresponding author; *email address:* dzampieri@units.it *(D. Zampieri)*

Table of contents

-Chemistry:

-^{1}H-NMR and ^{13}C-NMR Spectra for compounds **4a–j**

-Biology:

-Cytotoxicity curves for compounds **4d**, **4e**, **4g** and **4j**

-In silico:

-ADMET predicted profiles for compounds **4d, 4e, 4g** and **4j**.

^{1}H-NMR and ^{13}C-NMR spectra for compound **4a** ($CDCl_3$)

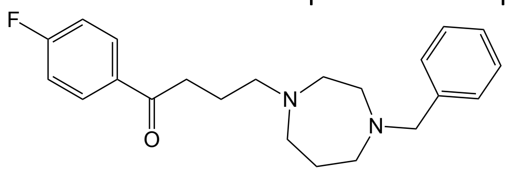

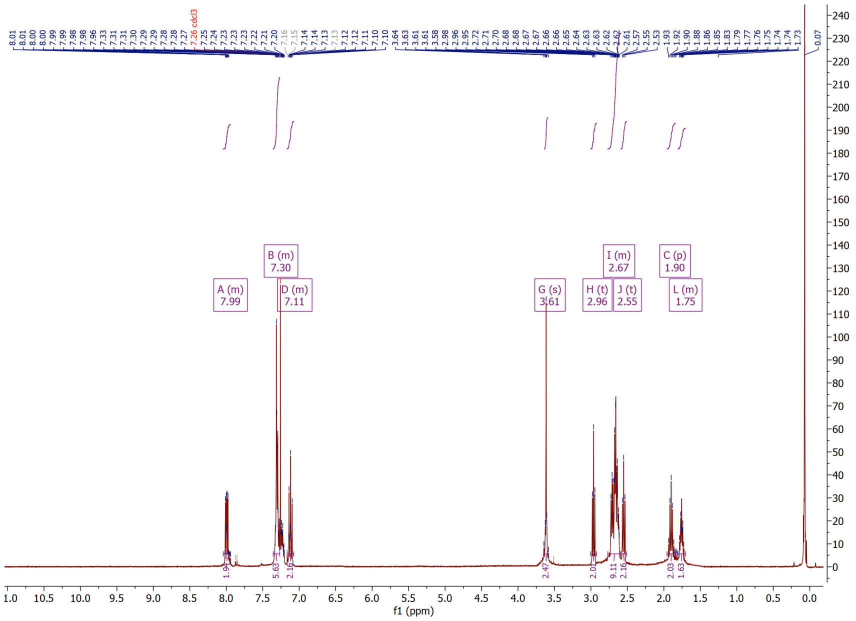

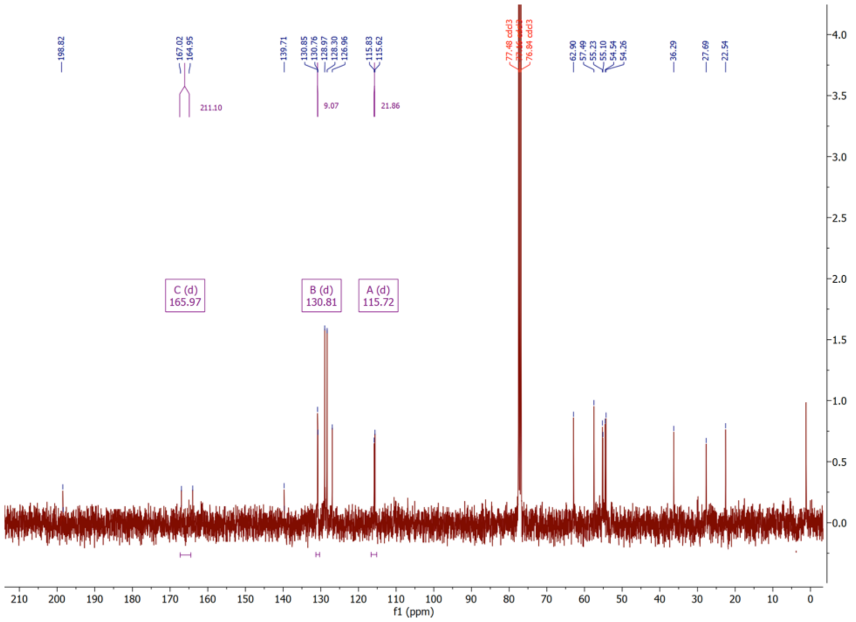

^{1}H-NMR and ^{13}C-NMR spectra for compound **4b** ($CDCl_3$)

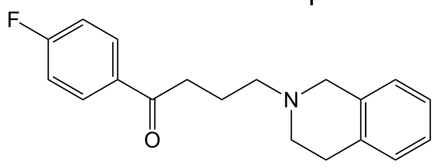

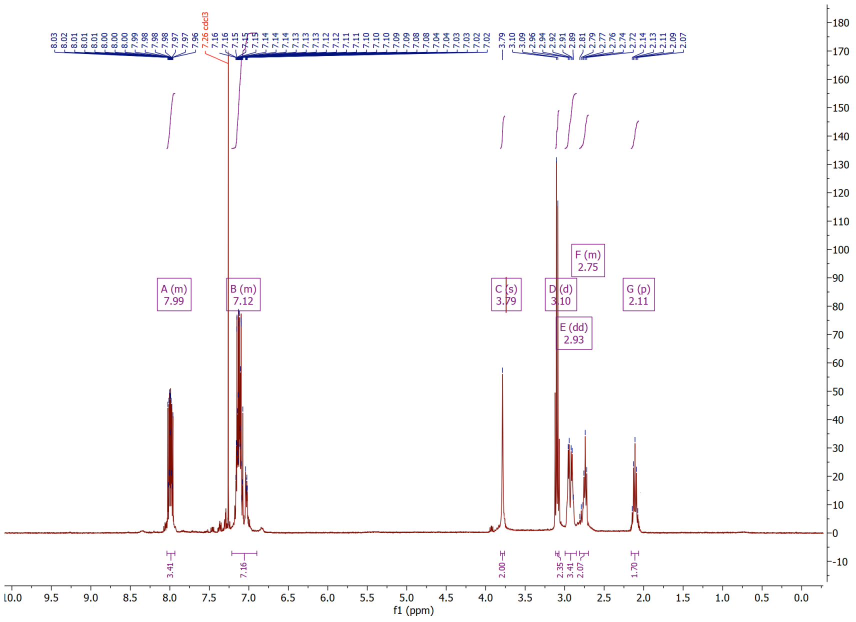

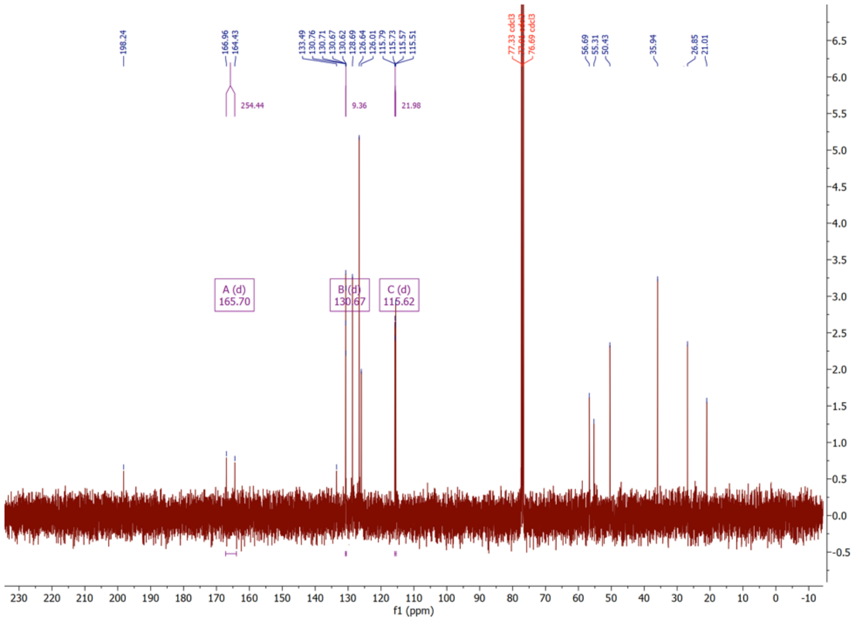

^{1}H-NMR and ^{13}C-NMR spectra for compound **4c** ($CDCl_3$)

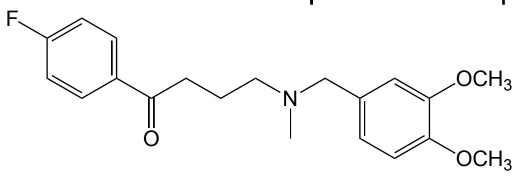

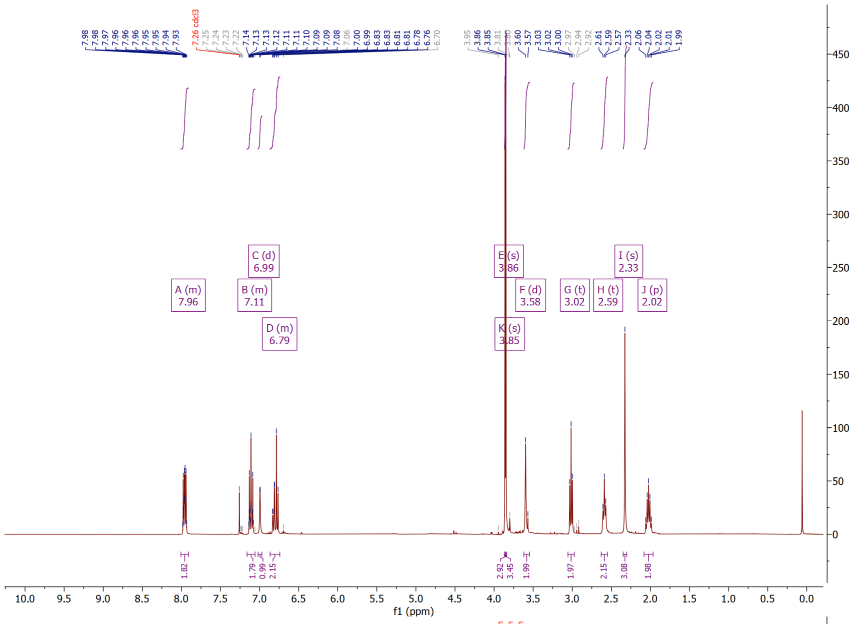

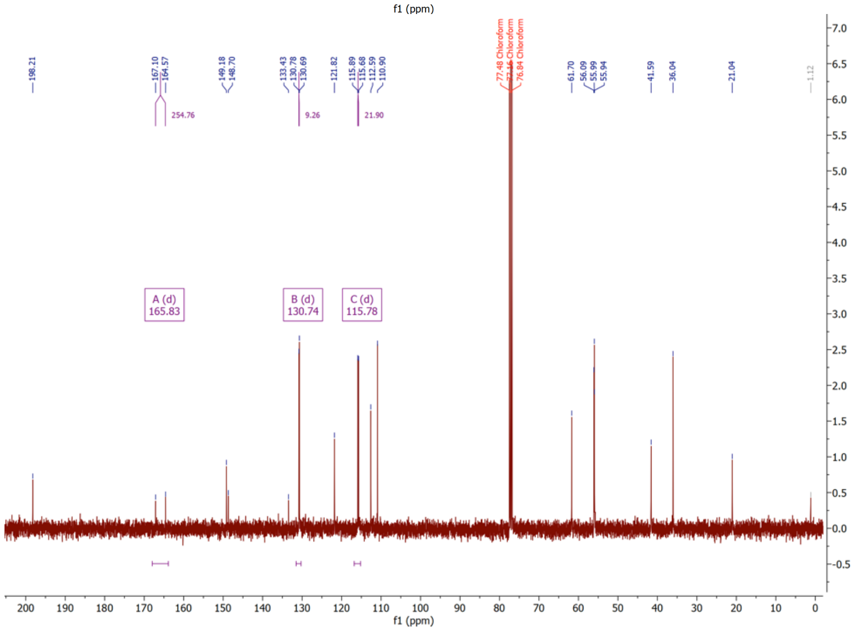

^{1}H-NMR and ^{13}C-NMR spectra for compound **4d** ($CDCl_3$)

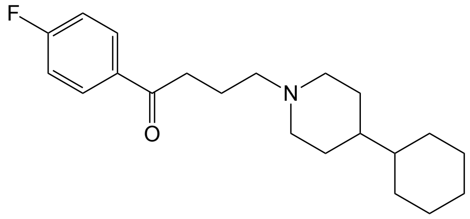

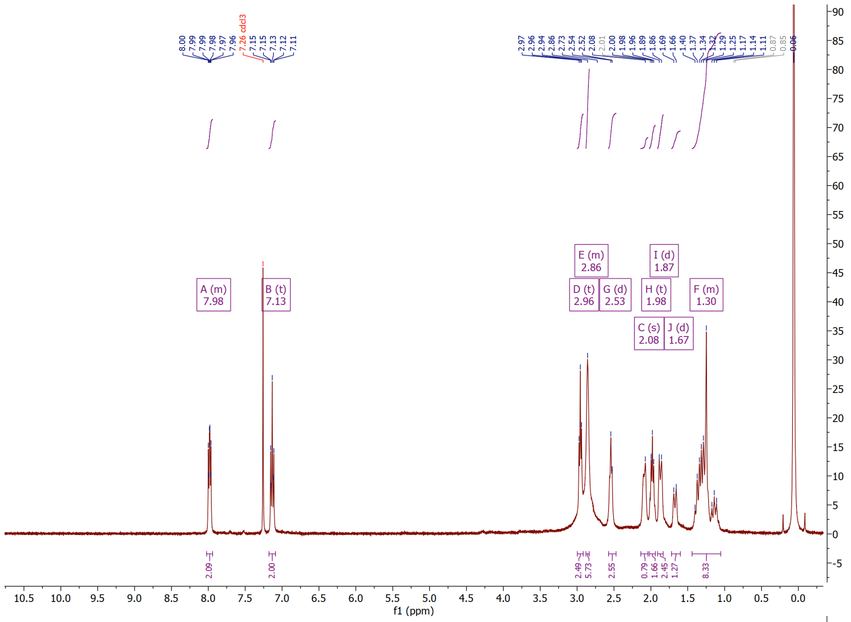

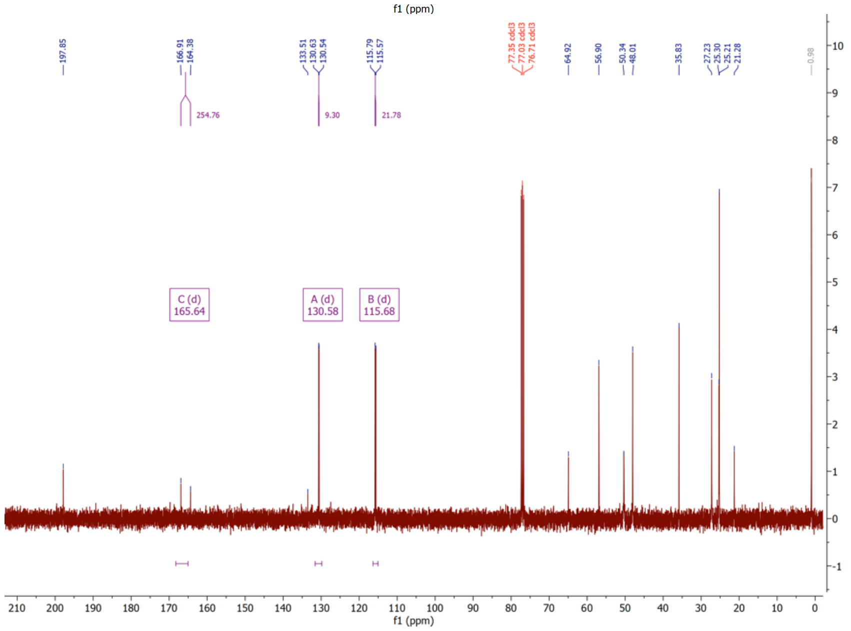

^{1}H-NMR and ^{13}C-NMR spectra for compound **4e** ($CDCl_3$)

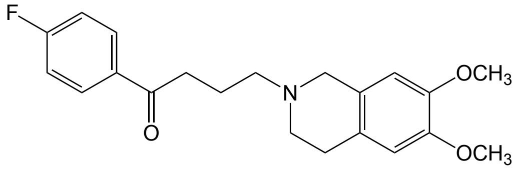

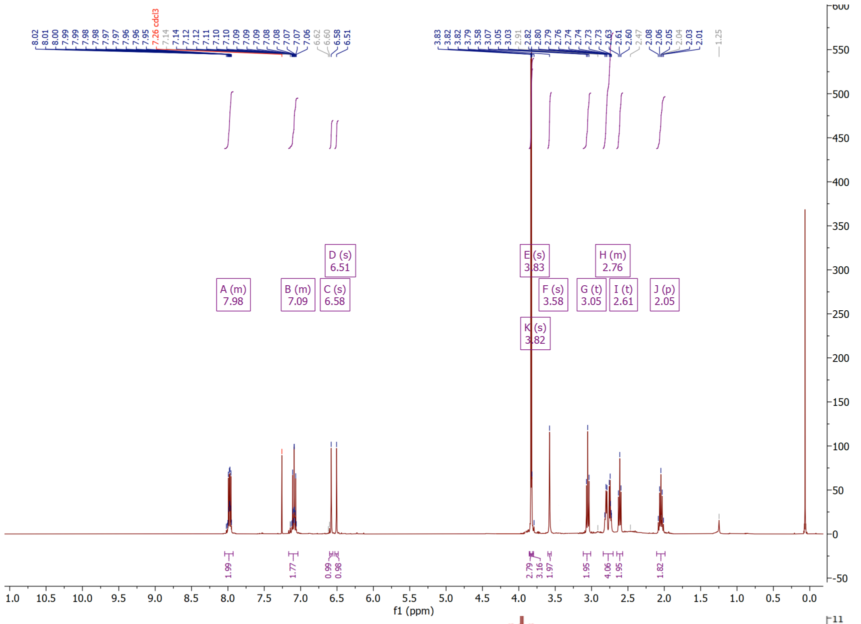

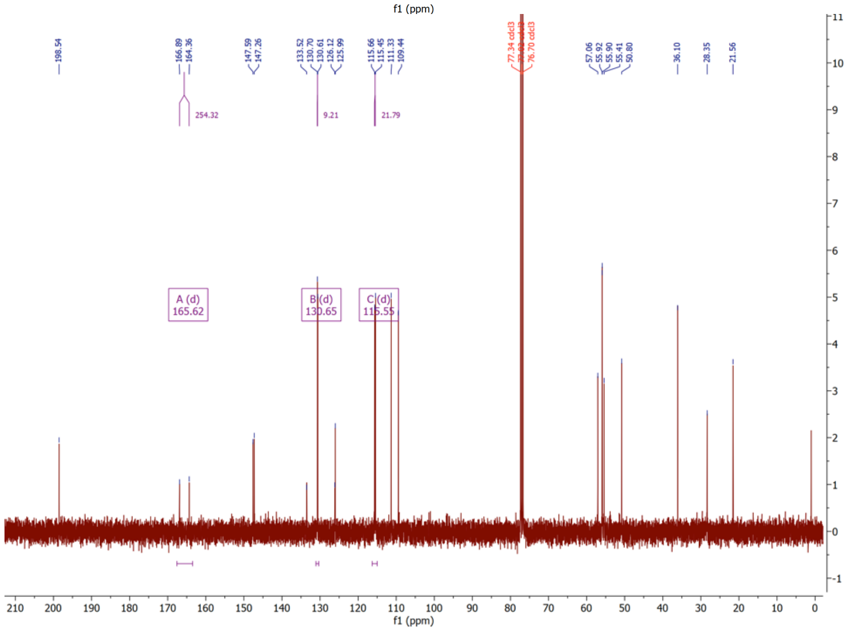

^{1}H-NMR and ^{13}C-NMR spectra for compound **4f** ($CDCl_3$)

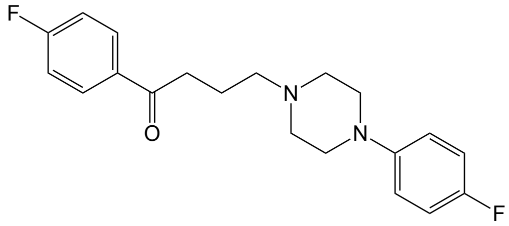

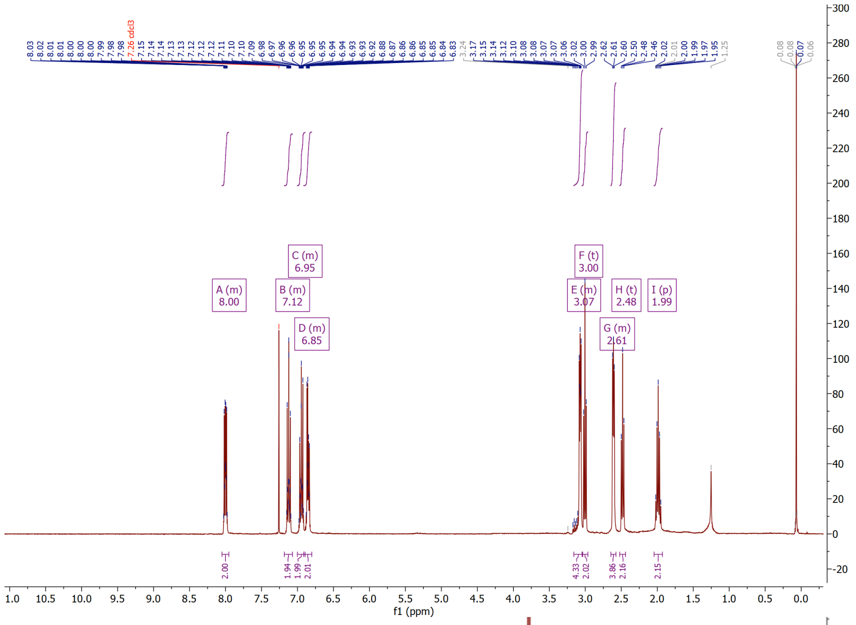

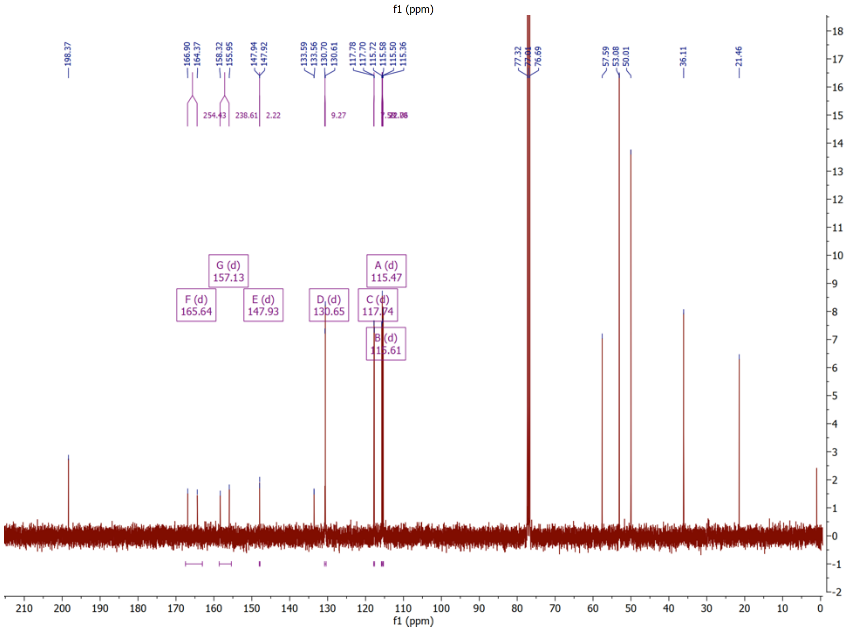

^{1}H-NMR and ^{13}C-NMR spectra for compound **4g** ($CDCl_3$)

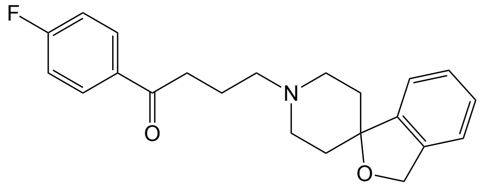

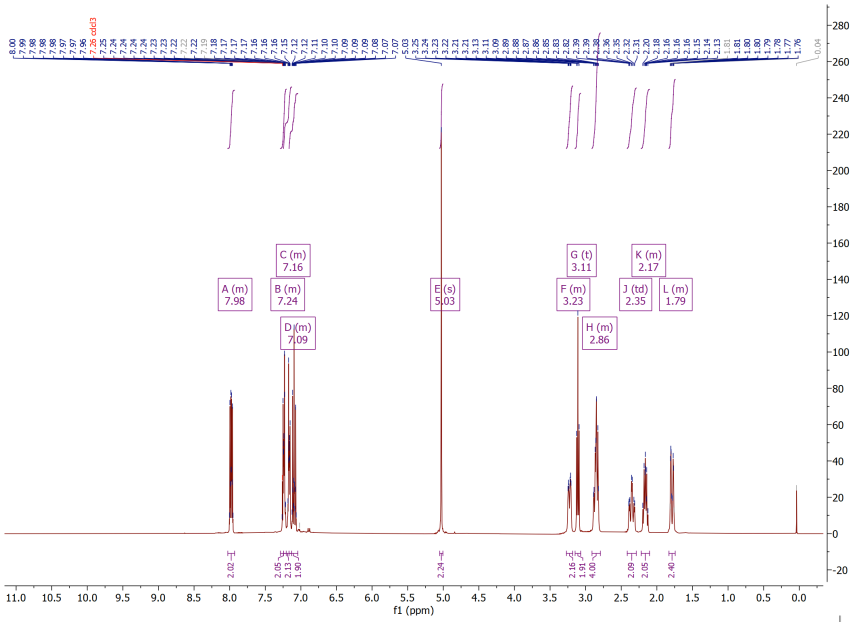

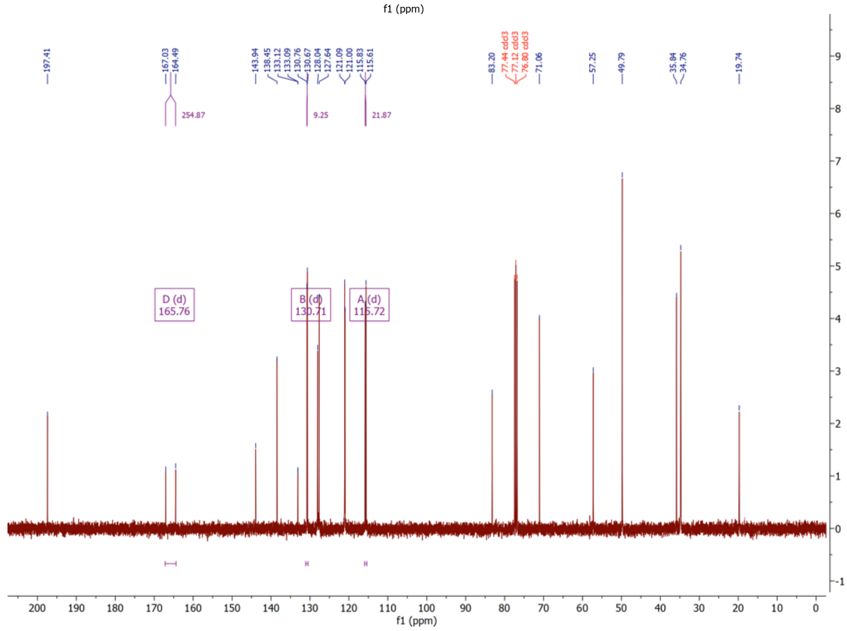

^{1}H-NMR and ^{13}C-NMR spectra for compound **4h** ($CDCl_3$)

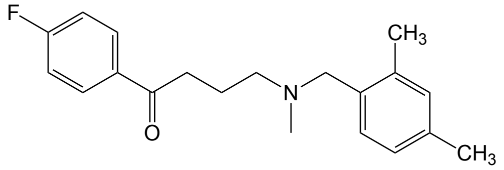

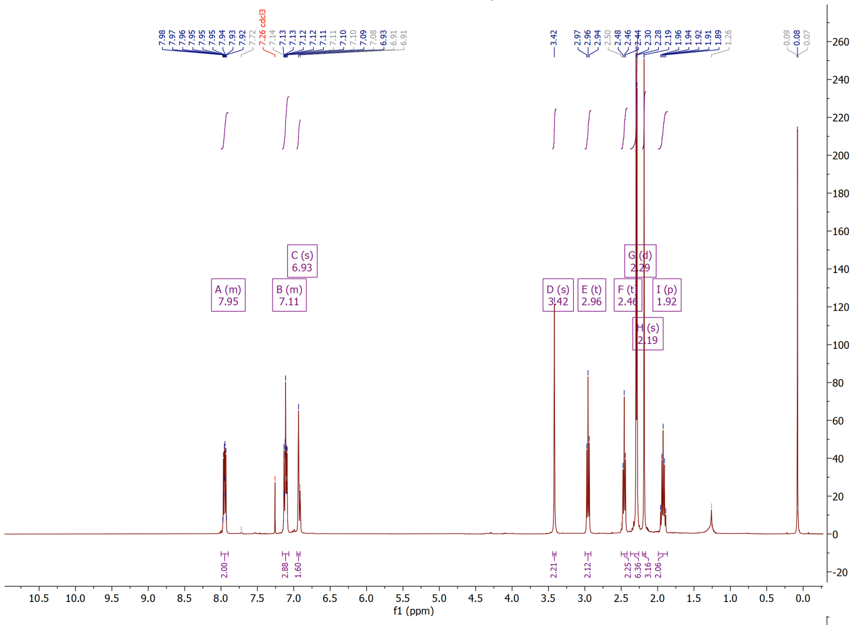

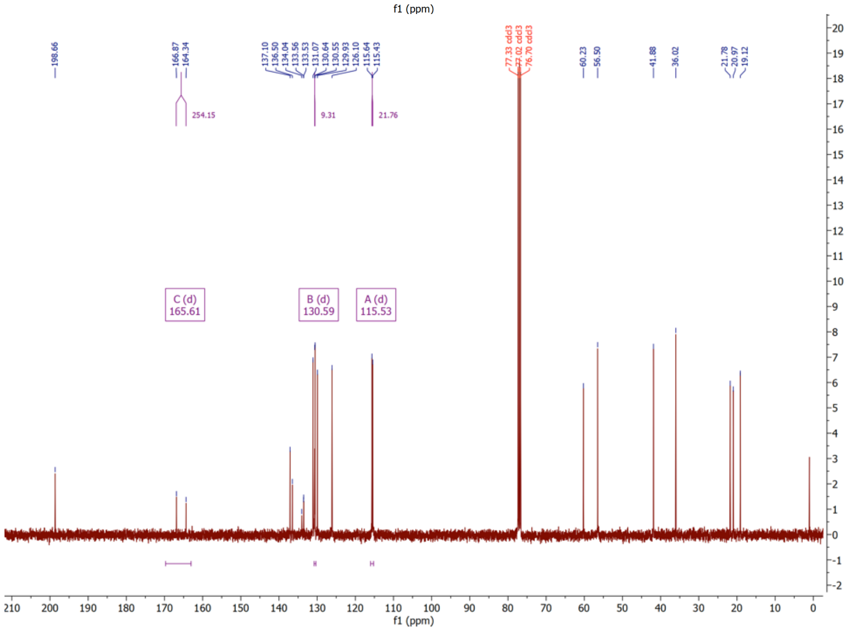

^{1}H-NMR and ^{13}C-NMR spectra for compound **4i** ($CDCl_3$)

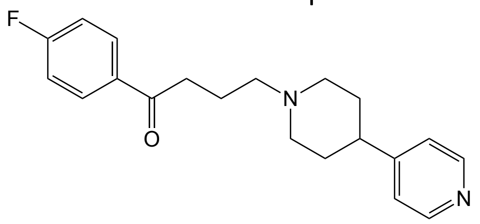

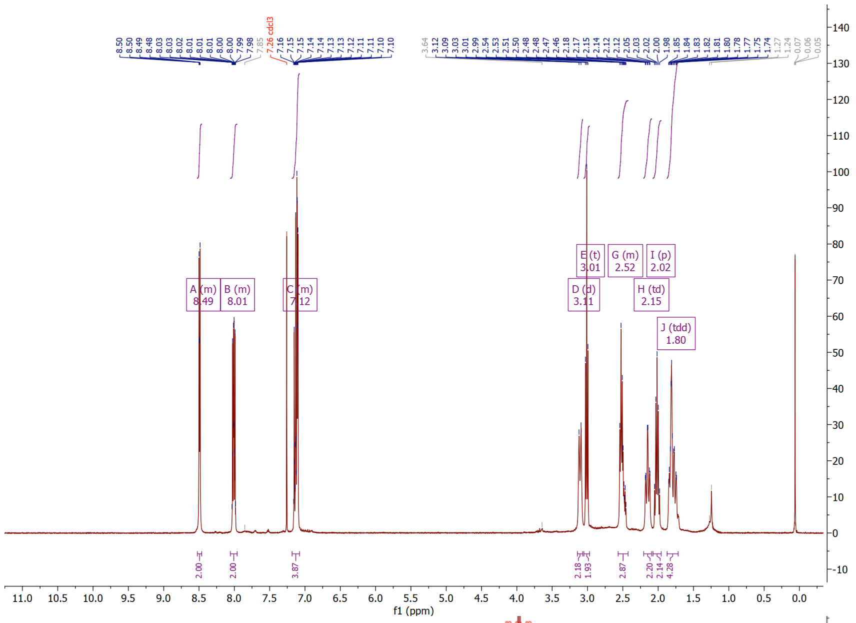

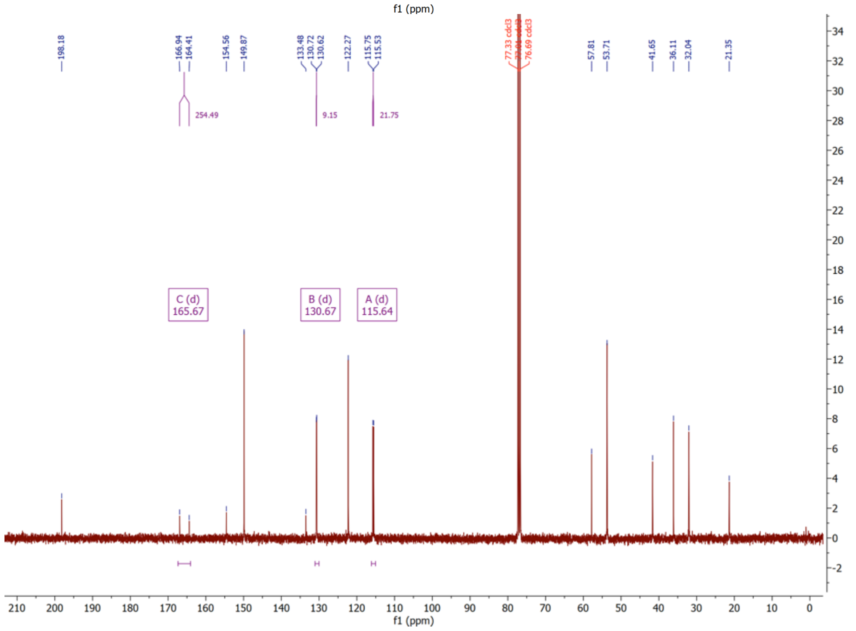

^{1}H-NMR and ^{13}C-NMR spectra for compound **4j** ($CDCl_3$)

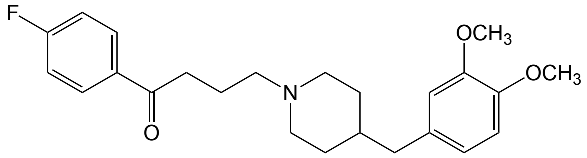

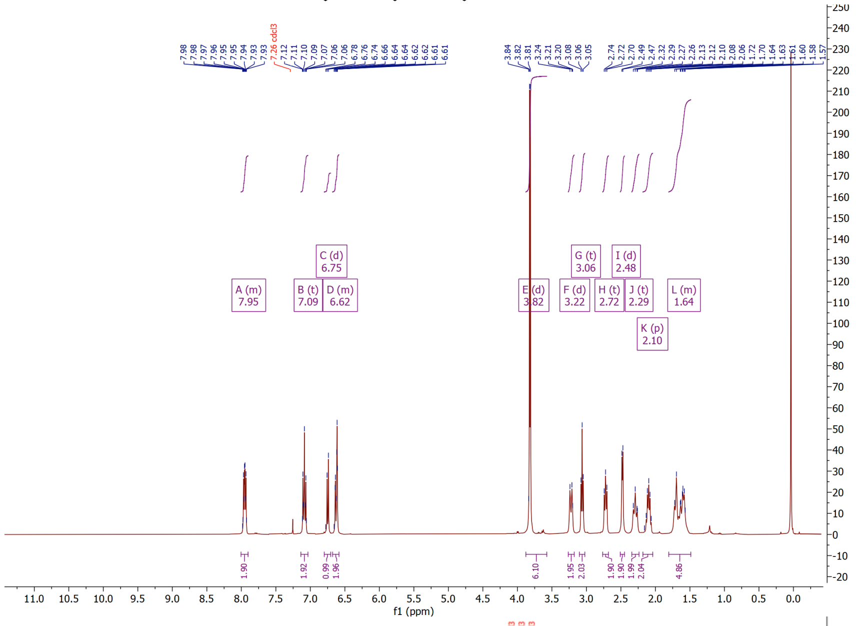

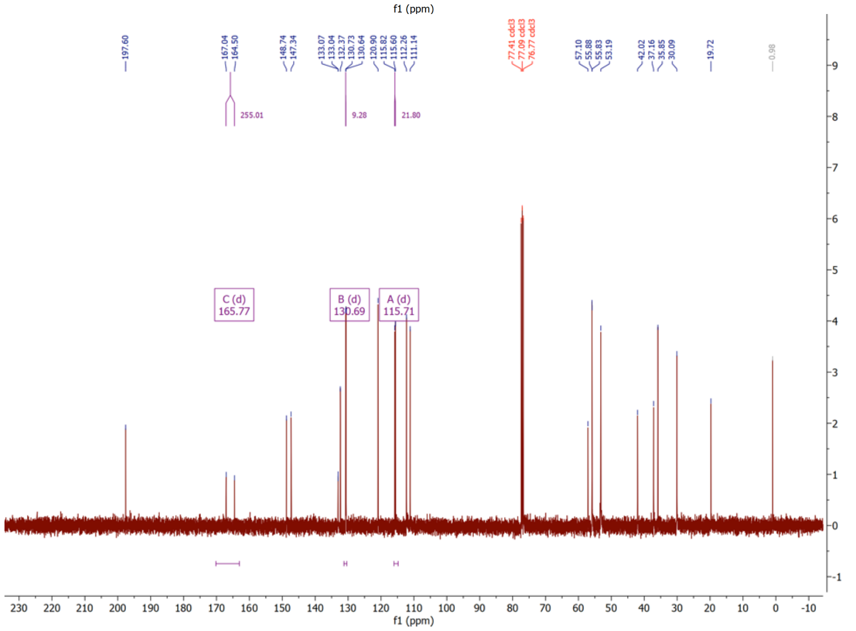

Figure S1. Cytotoxicity curves of novel SIGMAR1 ligands **4d, 4e, 4g,** and **4j** compared to reference compounds HAL and SRM, in SH-SY5Y cells. The x-axis shows the log10 molar concentration of each compound. The y-axis shows the percentage cytotoxicity relative to the DMSO vehicle control.

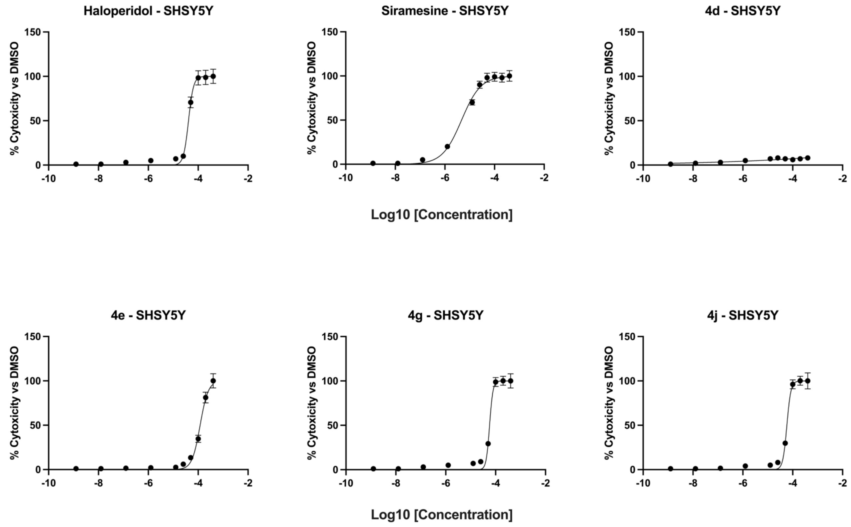

Figure S2. Cytotoxicity curves of novel SIGMAR1 ligands **4d, 4e, 4g,** and **4j** compared to reference compounds HAL and SRM, in HUH-7 cells. The x-axis shows the log10 molar concentration of each compound. The y-axis shows the percentage cytotoxicity relative to the DMSO vehicle control.

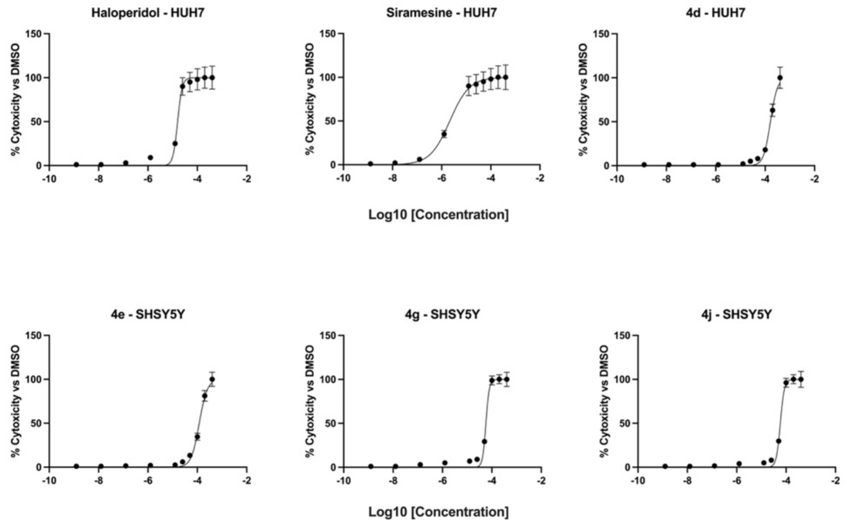

Table S1. Main ADMET predicted profile of novel SIGMAR1 ligands **4d, 4e, 4g,** and **4j** compared to reference compounds HAL and SRM.

Cmpd	carcinogenicity	Ames mutag.	Skin irritation	Epatotoxicity	Nephrotoxicity	Oral acute tox.	HERG inhib.
**4d**	–0.9300	–0.7500	–0.6959	+0.5081	–0.7577	(III) 0.7324	–0.4753
**4e**	–0.9300	–0.7700	–0.8034	–0.6000	–0.8447	(III) 0.6797	+0.8972
**4g**	–0.8938	–0.8300	–0.9734	–0.6875	–0.8986	(III) 0.6923	+0.9131
**4j**	–0.8700	–0.6100	–0.8126	+0.5500	–0.7766	(III) 0.6467	+0.7014
HAL	–0.7700	–0.9700	–0.6608	+0.5274	–0.8067	(II) 0.7338	–0.5000
SRM	–0.8600	+0.5500	+0.7890	+0.5625	–0.8128	(III) 0.6324	+0.9553
